# Supplementary material for: KRAS-dependent sorting of miRNA to exosomes
Source: eLife. 2015 Jul 1;4:e07197. doi: 10.7554/eLife.07197 (PMC4510696; doi:10.7554/eLife.07197)
Supplement: Figure 1—source data 1. — Small RNA sequencing libraries were prepared from three isogenic CRC cell lines with the indicated alleles of KRAS. Table is based on work done in Demory Beckler et al. (2013). DOI: http://dx.doi.org/10.7554/eLife.07197.004 [file elife07197s001.docx]

**Figure 1-source data 1**

| Cell line | *KRAS* allele | Growth in soft agar | Tumors in nude mice |
| --- | --- | --- | --- |
| DLD-1 | WT/G13D | Yes | Yes |
| DKs-8 | WT | No | No |
| DKO-1 | G13D | Yes | Yes |

**Figure 1-source data 1. Colorectal cancer cell lines.**

Small RNA sequencing libraries were prepared from three isogenic colorectal cancer cell lines with the indicated alleles of KRAS. Table is based on work done in ^21^.
