# Supplementary material for: KRAS-dependent sorting of miRNA to exosomes
Source: eLife. 2015 Jul 1;4:e07197. doi: 10.7554/eLife.07197 (PMC4510696; doi:10.7554/eLife.07197)
Supplement: Supplementary file 3. — Related to experimental procedures. Primers used for plasmid construction. DOI: http://dx.doi.org/10.7554/eLife.07197.025 [file elife07197s004.docx]

**Supplemental File 3**. Related to Experimental Procedures. Primers used for plasmid construction.

|  | Sequence 5’ - 3’ |
| --- | --- |
| mTOR_F_SpeI | actagtAGGCTTGATTTGGTTCCCAG |
| mTOR_R-HinD | aagcttAGTTGAGTATTTGTTCTGCTCA |
| miR-100-PT_F1 | ctagtTAA**cacaagttcggatctacgggtt**CGAT**cacaagttcggatctacgggtt**ACGC |
| miR-100-PT_F2 | GT**CACAAGTTCGGATCTACGGGTT**TCAC**CACAAGTTCGGATCTACGGGTT**A |
| miR-100-PT_R1 | **AACCCGTAGATCCGAACTTGTG**ATCG**AACCCGTAGATCCGAACTTGTG**TTAA |
| miR-100-PT_R2 | agctt**AACCCGTAGATCCGAACTTGTG**GTGA**AACCCGTAGATCCGAACTTGTG**ACGCGT |
| MS1_F | atgagttctcttgtgagttaagtcaaaaactgattttctaaagttatggatcttctgttccc |
| MS1_R | gggaacagaagatccataactttagaaaatcagtttttgacttaactcacaagagaactcat |
| MS2_F | ctgtgctgagtttgctgttctgatgttgagaggagcaactaggt |
| MS2_R | acctagttgctcctctcaacatcagaacagcaaactcagcacag |
| MS3_F | ctgtcatggaggtgctgaacacagggtcagtctagtacacattgtaaattatgagcagaacaaatactcaa |
| MS3_R | ttgagtatttgttctgctcataatttacaatgtgtactagactgaccctgtgttcagcacctccatgacag |

Restriction sites in lowercase. miR-100 sites in bold.
